# Supplementary material for: Design and experiments with a single spiral fixed depth ditching and fertilizing machine
Source: Sci Rep. 2023 May 13;13:7798. doi: 10.1038/s41598-023-34464-6 (PMC10182980; doi:10.1038/s41598-023-34464-6)
Supplement: Supplementary file 1 — Supplementary Information. [file 41598_2023_34464_MOESM1_ESM.docx]

Article

Design and experiments with a single spiral fixed depth ditching and fertilizing machine

Caixue Zhan ^1^, Wenqin Ding^1^, Yu Han^1^, Qinghai Jiang^1^, Ying Zhao^1^, Liang Zhao^2^and Zhiyu Song^1,^*

1. Nanjing Institute of Agricultural Mechanization, Ministry of Agriculture and Rural Affairs, Nanjing 210014, China; zhancaixue@caas.cn (C.Z.); [dingwenqin@caas.cn](mailto:dingwenqin@caas.cn) (W.D.); hanyu@caas.cn (Y.H.);

[jiangqinghai@caas.cn](mailto:jiangqinghai@caas.cn) (Q.J.); zhaoying@caas.cn (Y.Z.)

1. College of Mechatronics Engineering, Nanjing Forestry University, Nanjing 210037, China; [liangzhao123@njfu.edu.cn](mailto:liangzhao123@njfu.edu.cn) (L.Z.);

***** Correspondence: songzhiyu@caas.cn; Tel.: +86-25-8434-6239

**Abstract:** Aiming at the problems of low fertilization efficiency, mainly the process operation and inconsistent fertilization depth of domestic tea garden fertilizer machines, a single-spiral fixed depth ditching and fertilizing machine is appropriately designed. This machine is capable of performing the integrated operation of ditching, fertilization, and covering soil at the same time through the operation mode of single-spiral ditching and fertilization. The theoretical analysis and design of the structure of the main components are properly carried out. The fertilization depth can be adjusted through the established depth control system. The performance test reveals that the single-spiral ditching and fertilizing machine exhibits a maximum stability coefficient of 96.17% and a minimum of 94.29% in terms of trenching depth and a maximum of 94.23% and a minimum of 93.58% in terms of fertilization uniformity, meeting the production requirements of tea plantations.

1. Introduction

Fertilizer application is one of the crucial aspects of crop production, the quality of fertilizer application directly influences the growth of crops, and reasonable fertilizer application is commonly regarded as one of the important measures to ensure high quality and high yield of crops [1-4]. The main method of ditching and fertilizing in China is to build a trench with a rotary tiller, then utilize a fertilizer spreader or spread the fertilizer manually, and finally do soil tillage by hand [5-7]. This fertilization approach tends to have relatively low fertilizer use efficiency and residual fertilizer left on the surface, which could easily pollute the environment [8,9]. A suitable fertilization situation is able to improve the fertilizer utilization ratio [10,11]. A fertilization position that is too far from the roots could easily lead to poor crop absorption and a low fertilizer ratio, and a fertilization position that is too close to the roots could easily result in root burning fertilizer problems. Research by agronomists has revealed that a reasonable fertilization position ensures maximum absorption of fertilizer by crop roots, improves fertilizer utilization ratio and is crucial for lessening the fertilizer rate [12-15].

Foreign developed countries have the early start of research on fertilizing machines, and the development of their ditching machines has led to the rapid development of ditching and fertilizing devices, experiencing several stages of share plows, rotary ditchers, and chain ditchers [16-18]. The start of ditching and fertilizing machines was relatively late in the country, with rotary furrow fertilizing machines being mainly used [19]. Xiao et al. [20] developed a double spiral ditch and fertilizer machine for gardens, which is used in conjunction with a tractor to apply fertilizer evenly and can meet the agronomic needs of garden fertilization. Gaomi Yifeng Machinery Co., Ltd. produced a self-contained multi-purpose fertilization device, which passes through a screw fertilizer distributor to adjust the amount of fertilizer planting and a crawler chassis with good passing performance. Shen Congju et al. [21] developed a self-propelled gas deep loosening fertilization device for gardens, which solved the technical problems of fertilizer punching and dosing in hard ground, and a theoretical basis and technical support for the development of a gas explosion deep loosening fertilizing machine for orchard [21].

From the results of research works inside and outside the country, it is clear that domestic fertility machines are mainly one-process operations and lack automatic depth adjustment devices. Foreign ditching and fertilizing machines are more advanced, but they are expensive and do not meet the operational needs of our country. In the present paper, a spiral fixed-depth ditching and fertilizing machine is designed, which could appropriately perform the integrated operation of ditching, fertilization, and covering soil, and can automatically adjust the fertilization depth to achieve constant depth fertilization, in order to enhance the operational efficiency and fertilization effect of the ditching and fertilizing machine. This article is divided into four parts, where the first part gives an introduction to the current state of research on fertilizing machines. The second part is aimed to design the mechanical structure and control system of the fertilizing machine. The third part deals with the ditch test, fertilization test, and field test. The fourth part summarizes the whole article and the main obtained results.

2. Materials and Methods

**2.1. Complete machine structure and working principle**

**2.1.1. Complete machine structure**

**
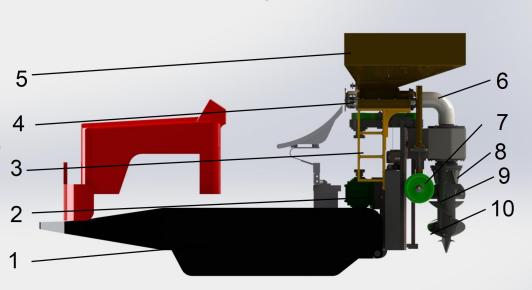
**In order to improve the fertilization efficiency, along with the agricultural needs of tea planting, a single-spiral fixed depth ditching and fertilizing machine is appropriately designed, where the corresponding whole structure is schematically illustrated in Figure 1. It essentially consists of a tractor, transmission mechanism, fertilizer box frame, screw fertilizer distributor, fertilizer box, fertilizer delivery pipe, fixed depth ground wheel, grass scraper, ditching and fertilizing mechanism, spiral blade, etc. The main technical parameters are also presented in Table 1.

**Figure 1.** Schematic representation of the fertilizer construction, whose main parts are itemized as follows: 1. Tractor, 2. Transmission mechanism, 3. Fertilizer box frame, 4. Screw fertilizer distributor, 5. Fertilizer box, 6. Fertilizer delivery pipe, 7. Fixed depth ground wheel, 8. Grass scraper, 9. Ditching and fertilizing mechanism, and 10. Spiral blade.

**2.1.2. Working principle**

The depth of the ditching is predetermined according to the agronomic requirements of fertilization before working the ditching and fertilizing machine operates. During ditching and fertilization, the tractor pulls the ditching and fertilizing device forward, the ditching and fertilizing machine cuts into the soil, the fertilizer is discharged through the screw fertilizer distributor and transported to the ditching and fertilizing mechanism through the fertilizer delivery pipe, and finally falls into the furrow cut by the ditching and fertilizing mechanism. At the same time, the soil falls back to complete the covering soil, realizing the integrated action of ditching, fertilizing, and covering the soil.

**Table 1.** Main technical parameters of the machine.

| Item | Description/Value |
| --- | --- |
| Structure type | Self-propelled (crawler) |
| Overall dimensions (length×width×height) (mm) | 2780×800×1600 |
| Engine type | Diesel engine |
| Maximum fertilization depth (mm) | 450 |
| Maximum ditching width (mm) | 250 |
| Fertilizer box volume (m^3^) | 0.3 |
| Operating speed (km/h) | 0.4-1.8 |

**2.2. Structure of the screw fertilizer distributor**

The fertilizer apparatus adopts the screw fertilizer distributor, which essentially consists of a fertilizer box, fertilizer outlet, fertilizer inlet, rotating shaft, and fertilizer spiral blade, as demonstrated in Figure 2.

**
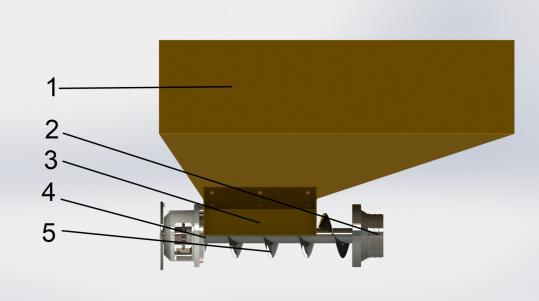
Figure2.** Schematic representation of the screw fertilizer distributor, whose main constituents are as follows: 1. Fertilizer box, 2. Fertilizer outlet, 3. Fertilizer inlet, 4. Rotating shaft, and 5. Fertilizer spiral blade.

**2.2.1. Diameter of the screw fertilizer distributor**

The diameter of the screw fertilizer distributor is one of the crucial factors of screw fertilizer distributors, which directly affects the amount of fertilizer planted by the screw fertilizer distributor and the uniform variation coefficient of fertilizer distribution [22]. Since the amount and speed of fertilizer planting are not taken into account, the formula for evaluating single-ring sowing-fertilizer takes the following form:

$\text{q}=\left[\frac{\pi\left(D^{2}-\text{d}^{2}\right)\text{ S}}{4}\text{-bh}L\right]\rho\phi$ （1）

$L=\sqrt{\left[\pi(D+\text{d）}/2\right]^{2}+S^{2}}$ （2）

$\text{h}=\text{（}D\text{-d）}/2$ （3）

where *D* represents the outer diameter of the screw fertilizer distributor (mm), *d* denotes the inner diameter of the screw fertilizer distributor (mm), *s* is the pitch of the screw fertilizer distributor (mm), *b* signifies the average thickness of the screw tooth of the screw fertilizer distributor (mm), *h* represents the depth of the screw tooth of the screw fertilizer distributor (mm), *L* is the average length of the screw tooth of the screw fertilizer distributor (mm), *ρ* denotes the fertilizer volume weight (g/mm^3^), and φ stands for the filling coefficient of the screw fertilizer distributor.

By virtue of Eq. (1), it can be seen that the single-ring fertilizer sowing amount (*q*) depends on various factors, including *D*, *d*, *S*, *ρ*, and *φ*. The single-ring fertilizer sowing amount (*q*) changes by altering the values of *D*, *d*, and *S*. The relationship between the outer diameter of the screw fertilizer distributor and the fertilizer sowing amount in the entire fertilizer sowing operation is given by:

$D=K\left(\frac{Q}{\phi\lambda\varepsilon}\right)^{\frac{2}{5}}$ （4）

$K=\left(\frac{1}{47\text{cA}}\right)^{\frac{2}{5}}$ （5）

where *Q* represents the fertilizer sowing amount of screw fertilizer distributor (t/h), *A* is the material comprehensive characteristic coefficient, *K* denotes the material comprehensive coefficient, *c* is the proportional coefficient of pitch to diameter, *λ* represents the mass of the material unit volume (t/m^3^), and *ε* denotes the transfer coefficient.

When the machine is in continuous operation, the fertilizer rate can be expressed by:

$Q_{S}=\text{v}_{\text{m}}\text{g}/\text{s}$ （6）

where *Q_s_* represents the fertilizer rate (t/h), *g* is the fertilizer rate to travel a distance (t), and *s* stands for the distance traveled (m).

According to the agronomic requirements for the fertilization of tea plantations, the amount of fertilization required for a tea plant is predicted to be 1.8 kg. Then one row of tea trees needs the amount of fertilizer for 0.9 kg, and the plant spacing of tea trees is generally 1.5-1.8 m. By choosing an average speed of 1100 m/h for the travel speed of the fertilizer machine, the fertilizer rate (*Qs*) can be obtained as 0.6t/h and the fertilizer sowing amount (*Q*) is also predicted to be 0.6t/h on the basis of Eq. (6). Along with the parameters of the fertilizer material, it can be determined that the filling coefficient of the fertilizer (*φ*) is 0.25, the comprehensive characteristic coefficient of the fertilizer (*A*) is 28, the unit mass of the material of the fertilizer is 1.2 T/m^3^, the proportional coefficient of the pitch to diameter is 0.9, and the conveying coefficient is 0.9, which are substituted into Eqs. (4) and (5). Further calculations reveal that the outer diameter of the screw fertilizer distributor would be 91 mm. Since such a factor should be designed as a standard series, its value can be also taken as 88 mm.

**2.2.2. Pitch of the screw fertilizer distributor**

The pitch of the screw fertilizer distributor determines the angle of ascent of the screw and the speed of fertilizer progress, which exhibits a crucial impact on the amount and uniformity of fertilizer from the screw fertilizer distributor [24], and the pitch of the screw fertilizer distributor is evaluated per the following relation:

$S=K_{1}D$ (7)

where *K_1_* signifies the proportional coefficient of the pitch and diameter, usually in the range of 0.8-1.0, which is mostly related to the flow ability of the fertilizer.

According to the existing design experience, comprehensive analysis to take *K_1_*=0.8, so the pitch is 70.4mm, rounded to 70mm.

2.2.3. Rotational speed of the screw fertilizer distributor

In order to maximize the fertilization uniformity while meeting the fertilizer requirements, the suitable rotational speed of the rotating shaft can ensure the stability of the fertilizer rate. It should be noticed that the rotational speed of the screw fertilizer distributor can be determined based on the fertilizer rate, the diameter of the screw fertilizer distributor, and the relevant material parameters of the fertilizer to determine the better rotational speed [25]. When the rotational speed is too high, the fertilizer will stick to the inner wall of the screw fertilizer distributor under the action of centrifugal force; therefore, the centrifugal force of the fertilizer and gravity should satisfy the following requirements:

$m\omega^{2}R\leq mg$ （8）

$\text{m（}\frac{2\pi\text{n}_{\text{max}}}{60}\text{）}^{2}R\leq\text{mg}$ （9）

$\frac{\pi\text{n}_{\text{max}}}{30}R\leq\sqrt{\text{g}R}$ （10）

Considering the effect of various fertilizers,

$\frac{\pi\text{n}_{\text{max}}}{30}R\leq K{}_{0}\sqrt{\text{g}R}\text{（}0\leq K_{0}\leq1\text{）}$ （11）

$\text{n}_{\text{max}}=\frac{30K_{0}}{\pi}\sqrt{\frac{\text{g}}{R}}=\frac{30K_{0}\sqrt{2\text{g}}}{\pi\sqrt{D}}$ （12）

$A=\frac{30K_{0}\sqrt{2\text{g}}}{\pi}$ （13）

$\text{n}_{\text{max}}=\frac{A}{\sqrt{D}}$ （14）

where *A* represents the fertilizer comprehensive coefficient, *K_0_* denotes the fertilizer comprehensive characteristic coefficient, *n_max_* is the maximum speed of the screw fertilizer distributor (r/min), and *n* stands for the rotational speed of the crew fertilizer distributor (r/min). The fertilizer sowing amount of the crew fertilizer distributor can be calculated as:

$Q=\frac{\pi\left[\left(D+2\lambda\right)^{2}-\text{d}^{2}\right]}{4}60\phi S\text{npc}$ （15）

$\text{n}=\frac{Q}{15\pi\phi\rho S\text{c}\left[\left(D+2\lambda\right)^{2}-\text{d}^{2}\right]}$ （16）

According to Eqs. (14) and (16), the rotational speed of the crew fertilizer distributor should meet the following condition: *n*≤200 (r/min).

**2.3. Spiral ditching and fertilizing structure**

**
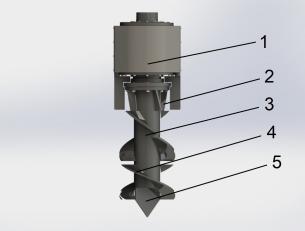
**The spiral ditching device of the single-spiral ditching and fertilizing machine uses single-spiral ditching, as demonstrated in Figure 3. It is mainly composed of a transmission case,baffle plate,cutter,spiral blade,and tool bit. The way of single-spiral ditching has a simple and close-fitting structure,low consumption, and the edge part of the spiral blade incorporates into an increase of the soil cutting blade, which could effectively prevent the development of extra deformation.

**Figure 3.** Structure schematic diagram of the spiral ditching and fertilizing, whose main constituents are as follows: 1. Transmission case, 2. Baffle plate, 3. Cutter arbor, 4. Spiral blade, and 5. Tool bit.

**2.3.1. Diameter of the spiral blade and tool bit**

The single-spiral ditching and fertilizing machine is driven in tea fields, relying on the spiral blade for ditching operation, and together with the relevant agricultural needs, the ditch width is determined to be not less than 250 mm. The diameter of the spiral blade is:

$D_{1}=\text{（}0.92\sim0.98\text{）}D_{2}$ （16）

where *D_1_* represents the diameter of the spiral blade (mm), and *D_2_* denotes the ditch width of the fertilizing machine (mm).

The diameter of the spiral blade is selected as 250 mm since the single-spiral and fertilization machine requires a maximum ditch depth of 450 mm. Further, the height of the spiral blade is set as 350 mm, and the height of the tool bit is adjusted to 100 mm because the spiral blade should accomplish the cutting and slanting operation to the soil. At the same time, it performs the straight line ditching operation, indicating the high necessity for high strength and hardness of the material; hence, CR12MOV with a thickness of 10 mm is chosen as the material.

**2.3.2. Establishment of** **the spiral curve equation of spiral blade**

The spiral blade is the main parameter of the spiral ditching and fertilizing mechanism, and its structural parameters could directly influence the quality and power consumption of ditching [26-28]. The single-spiral ditching and fertilizing machine employs a cylindrical variable spiral ditching mechanism, and the variable pitch spiral curve consists of numerous tangents (see Figure 4).


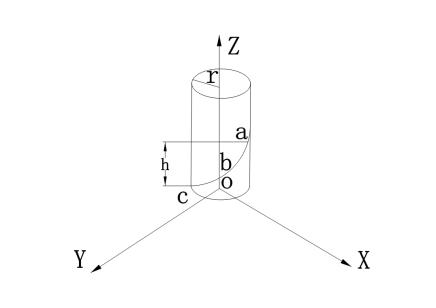

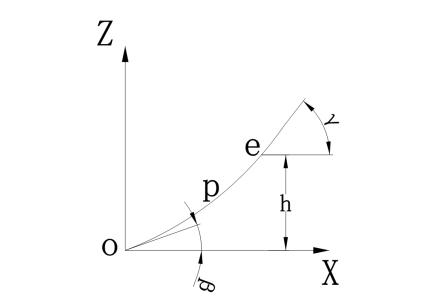


**Figure 4.** Schematic representation of the spiral curve.

Consider a point A on the generatrix along the *z*-axis, and regard that as a doing upward uniformly accelerated motion. Therefore, its trajectory forms a parabola on the *ZOX* plane, whose spiral curve can be expressed by the following relation:

$\left\{\begin{matrix} x=r\cos（2\pi rn） \\ y=r\sin（2\pi rn） \\ z=a\left(2\pi r\right)^{2}n^{2}+\left(2\pi r\tan\beta\right)n \end{matrix}\right.$ （17）

$\text{a}=\frac{\text{h-}2\pi\text{rn}_{1}\text{tanβ}}{\left(2\pi\text{rn}_{1}\right)^{2}}$ （18）

where *r* represents the radius of the ditching knife (mm), *n* denotes the number of spiral turns, *γ* signifies the spiral angle (°), *n_1_* is the number of spiral turns at B, β represents the spiral angle at C (°), and *h* denotes the height of point F (mm).

The efficiency of the spiral ditching and fertilizing mechanism relies on the action of the spiral blade to complete the cutting and lifting operation on the soil. According to the agricultural needs of tea fertilization, the width of ditching is set in the range of 200-300 mm and the corresponding depth is considered to be 300-400 mm. Taking into account the power consumption, as well as the depth and width of fertilization, the main parameters of the spiral ditching and fertilizing mechanism are designed by combining Eqs. (17) and (18), as presented in Table 2.

**Table 2.** Main technical parameters of the spiral ditching and fertilizing mechanism.

| Items | Value |
| --- | --- |
| Spiral blade diameter (mm) | 250 |
| Spiral blade height (mm) | 350 |
| Tool bit height (mm) | 100 |
| Spiral blade thickness (mm) | 10 |
| Initial spiral angle (°) | 35 |
| Maximum rotation speed (rad/s) | 5 |

**2.4. Control system design**

**2.4.1. Design of automatic adjustment device for fixed depth ditching mechanism**

The single-screw ditching mechanism cuts to the bottom of the ditch and its automatic adjustment process has been illustrated in Figure 5.


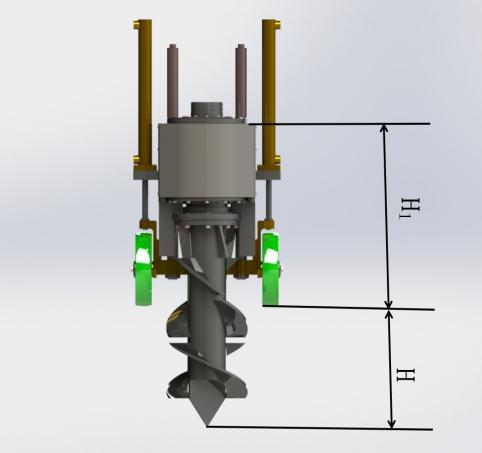
**Figure 5.** Schematic representation of the automatic depth adjustment.

During the ditching operation, the depth of the ditch and fertilization (*H*) is obtained by setting the height of the fixed depth (*H_1_*) according to the growth of tea trees and the agronomic requirements of fertilization. The ditching cutter arbor rotates and cuts into the soil, as the fixed-depth ground wheel touches the ground. At this time, the depth sensor of the fixed depth ground wheel transmits the signal to the single-chip microcomputer in real-time, which is responsible for controlling the expansion and contraction of the hydraulic cylinder. To this end, the relay and solenoid valve are mainly employed to realize the real-time adjustment of the ditching and fertilizing depth and keep that constant (i.e., *H*=cte). The adjustment principle diagram is provided in Figure 6.

**Figure 6.** Schematic chart of the depth automatic adjustment.

**2.4.2. Hardware and software design of automatic adjustment system for fixed depth ditching mechanism**

The fixed depth control system is the main control system of the spiral fixed depth ditching and fertilizing machine, which is mainly composed of a hardware system and a software system, which controls the depth of ditching and fertilizing through real-time feedback of the fixed depth ground wheel, and its control system has been demonstrated in Figure 7. The control system program is coded in C language. The main program calls several subroutines to perform control of the entire machine. The control system is based on STM32 L1 single-chip microcomputers as the core control element and is essentially composed of a power supply, voltage regulator module, and execution module. The power supply is from a 24V, 40AH lithium battery manufactured by Shenzhen Xinheng Power Technology Co., Ltd. It is used to provide an independent power supply for the entire control system and by utilizing the voltage regulator module, a stable power supply is provided for electronic components. The fixed depth ground wheel is mounted on the operating lever of the profile control valve (i.e., a Danfoss PMF profiling control valve with a maximum operating pressure of 28 bar), which is utilized to detect the heave of the fixed depth ground wheel in real-time and thus control the depth of ditching and fertilizing. The hydraulic motor is a BRM-50 from Foshan Hongpeng Hydraulic Co., Ltd. With a rotation speed of 750 rpm and a torque of 89 N.m, which is employed to control the rotation speed of the screw fertilizer distributor and thus the fertilizer rate.

**Figure 7.** Framework diagram of the control system of a single-spiral ditching and fertilizing machine.

**2.5. Test conditions and method**

The trial was conducted on March 19, 2022, in a standard tea plantation with a plant spacing of 300 mm and a row spacing of 1500 mm at Shenhang Forest, Tea and Fruit Seed Technology Co. The weather was clear, the temperature varied in the range of 3-15^o^, the wind speed was less than 3 m/s, the relative humidity of air was measured as 65%, the test land was loam, the absolute moisture content was 17.3%, and the testing ground was flat. The fertilizer chosen for the experiment was granular organic fertilizer produced by Hebei Dewaldo Fertilizer Co., Ltd. With a moisture content of about 1.5% and a diameter of about 3.3 mm, the test method adopts the methods stipulated in the Technical Specification of Quality Evaluation for Fertilizing Machinery (NY/T 1003-2006) and the Measuring Methods for Agricultural Machinery Testing Conditions-General Rules (GB/T 5262-2008).

3 . Results and Discussion

**3.1.** **Ditching test**

For the ditching test, the single-spiral ditching and fertilizing machine was operated at a uniform speed of 1.5 m/s to make three operational strokes, each 80 m long, with 10 m before and after the two stroke’s sections remaining as its reserved area, and to test 5 points equally between 20 and 70 m. The average ditching depth and the stability coefficient of each stroke are separately evaluated, and the test results are given in Table 3.

**Table 3.** The results of the ditching test.

| Parameters | Test stroke 1 | Test stroke 2 | Test stroke 3 |
| --- | --- | --- | --- |
| Set ditching depth (mm) | 250 | 300 | 350 |
| Maximum ditching depth (mm) | 259 | 311 | 358 |
| Minimum ditching depth (mm) | 243 | 294 | 249 |
| Average ditching depth (mm) | 250.3 | 299.3 | 299.5 |
| Ditching depth stability coefficient (%) | 94.81 | 95.33 | 96.3 |

The ditching test results indicate that the maximum difference in depth is 17 mm subjected to various strokes and the minimum value of the coefficient of stability of the ditching depth in the case of three strokes is 94.81%, which is in accordance with the evaluation index for the ditching machine operation.

**3.2.** **Fertilization test**

In the test tea garden, the single-spiral ditching and fertilizing machine performed under 6 strokes of fertilizing operation. For this purpose, the length of each operational stroke and the length of the protected area before and after was set equal to that of the ditching test, from a stroke of 10 m to a stroke of 40 m. In this interval, based on considering each 10 cm as a test interval, each stroke was divided into 30 test intervals, resulting in a total of 180 measurements. During the test, the single-spiral ditching and fertilizing machine moved at a uniform speed, and in the preparation stage, the spiral ditching and fertilizing mechanism raised and traveled through the test area at a constant speed. The fertilization in each area was then collected separately, and its weight was assessed by an electronic scale. After the completion of the individual travel operation, its fertilization uniformity was calculated in turn, and its appropriate test results are provided in Table 4.

Table 4. The results of the ditching test.

| Parameters | Test stroke 1 | Test stroke 2 | Test stroke 3 | Test stroke 4 | Test stroke 5 | Test stroke 6 |
| --- | --- | --- | --- | --- | --- | --- |
| Fertilization uniformity (%) | 94.21 | 95.66 | 93.15 | 96.35 | 95.12 | 93.33 |

Th Fertilization test results reveal that the fertilization uniformity for each individual stroke is higher than 93.15%, which meets the operational requirements of the evaluation index for the fertilizing machine.

**3.3. Field test**

Test site: The test was conducted in April 2022 at the tea garden of Pingshan Tea Factory in Liuhe District, Nanjing, China, and the field trial has been illustrated in Figure 8.

**
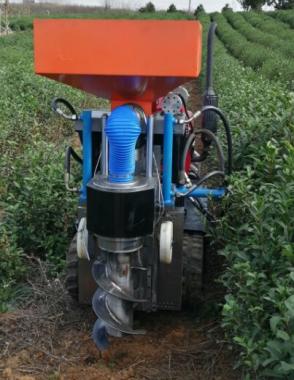
Figure 8.** Field test of the single-spiral ditching and fertilizing machine.

Test conditions: Sunny weather, temperature 4-22^o^C, breeze, plant spacing of 290 mm and row spacing of 1600 mm in the tea garden, flat land in the test tea garden, the soil is sandy loam, and soil moisture content of 13.2%.

Test materials: Granular organic fertilizer produced by Hebei Dewaldo Fertilizer Company.

Repeated tests were performed with three strokes and the test results are presented in Table 5. The experimentally observed data revealed that the coefficient of the ditching depth of the single-spiral ditching and fertilizing machine was at a maximum of 96.17% and a minimum of 94.29%. The maximum level of fertilization uniformity was reported as 94.23% and the minimum was obtained as 93.58%. These results meet the operational requirements of the evaluation index for the fertilizing machine.

**Table 5.** The results of the field test.

| Parameters | Test stroke 1 | Test stroke 2 | Test stroke 3 |
| --- | --- | --- | --- |
| Ditching depth stability coefficient (%) | 96.17 | 94.29 | 94.56 |
| Fertilization uniformity (%) | 93.65 | 94.23 | 93.58 |

4. Cconclusions

(1) In the current investigation, a single-spiral fixed depth ditching and fertilizing machine is designed, which can perform the integrated operation of ditching, fertilization, and covering soil, and can automatically adjust the fertilization depth to achieve a constant fertilizing depth. Through the theoretical analysis, the screw fertilizer distributor, the spiral ditching and fertilizing mechanism, and the corresponding control system are designed, and the ditching depth is automatically adjusted by the fixed depth control system, so that the operation efficiency and fertilization effect of the ditching and fertilizing machine were appropriately improved.

(2) The fertilizing machine designed in this paper can effectively solve the problem that the fertilization position of the traditional fertilizing machine is unreasonable, which readily makes the fertilizer use efficiency relatively low, and the residual fertilizer on the surface can easily pollute the environment. The fixed depth fertilization can be realized through the constant depth control system, so as to ensure the reasonable position of fertilization, which enhances the utilization rate of fertilizer. The fertilizing machine designed is able to effectively solve the problem of unreasonable fertilization position, improves the utilization rate of fertilizer, and exhibits significant impacts to reduce the fertilizer rate.

(3) The field test results revealed that the coefficient of stability of the ditch depth of the single-spiral ditching and fertilizing machine was at the maximum of 96.17% and at the minimum of 94.29%. The maximum fertilization uniformity value was also reported to be 23.94% and 93.58% at the minimum, meeting the requirements of tea plantation production.

**Author Contributions:** Conceptualization, C.Z. and W.D.; methodology, C.Z., W.D. and Y.H.; software, Y.H. and Y.Z.; validation, Z.S., Q.J. and L.Z.; formal analysis,C.Z. and W.D.; investigation, C.Z., W.D., Q.J. and Y.H.; resources, C.Z. and W.D.; data curation, C.Z. and W.D.; writing—original draft preparation, C.Z., W.D. and Q.J.; writing—review and editing,C.Z., W.D. and Q.J.; visualization,C.Z., Q.J. and W.D.; supervision, Y.H. and Z.S.; project administration, C.Z.; funding acquisition, Z.S. All authors have read and agreed to the published version of the manuscript.

**Funding:** Funding：This research was funded by Institute-level basic scientific research business expenses project of the Chinese Academy of Agricultural Sciences (Grant No.S202206),China Agriculture Research System of MOF and MARA(Grant NO. CARS-19),The Agricultural Science and Technology Innovation Project of the Chinese(Grant No. CAAS-ASTIP-2022-NIAM),Yunnan Province Key R&D Special Project(Grant No. 202102AE090038),Chengdu Agricultural Science and Technology Centre Local Financial Special Funds Project "Research and Development and Demonstration of Mechanized Operation Equipment for Hilly Orchards"(Grant No. NASC2020AR03),"Pioneer" and "Leading Goose" R&D Program of Zhejiang(Grant No. 2022C02010),Science and technology projects in Jiangsu Province(Grant No. BE2020315).

**Conflicts of Interest:** The authors declare no conflict of interest.

**Data availability:**The datasets used and/or analysed during the current study available from the corresponding author on reasonable request.

References

1. HERMAWAN W. Conceptual Design of Fertilizer Applicator for Oil Palm on Terrace Cultivation; proceedings of the 2nd International Conference on Agricultural Engineering for Sustainable Agricultural Production (AESAP), Bogor Agr Univ, Bogor, INDONESIA, F 2018Oct 23-25, 2017 . 2018.
2. FUADI M, SUTIARSO L, RADI R. Performance Test and The Implementation of Liquid Fertilizer Applicator based on Variable Rate Application (VRA) for Soybean (Glycine max) . Agritech, 2021, 41(2): 172-83.
3. OTAKE T, SATO M, SHINDO H, et al. POWER FARMING SYSTEMS FOR WELSH ONION CULTIVATION; proceedings of the 6th International Conference on Trends in Agricultural Engineering (TAE), Czech Univ Life Sci, Fac Engn, Prague, CZECH REPUBLIC, F 2019Sep 07-09, 2019 . 2019.
4. Zhao, L.; Zhou, H.;Xu, L.;Song, S.;Zhang, C.; Yu, Q.X. Parameter calibration of coconut bran substrate simulation model based on discrete element and response surface methodology. *Powder Technology*,**2022**, *395, 183-194.*
5. Zhan, C.;Ding, W.;Han, Y.;Jiang, Q.;Zhao, Y.;Zhao, L.; Song, Z. Design and experiments of an automatic depth-adjusting double screw trencher and fertiliserning.*PLOS ONE*,**2022**, *17 (12), e0277824.*
6. SUMON M M, MASHIUR R M, ARSHADUL H M, et al. Design and Performance Evaluation of NPK Briquette Applicator for Small-Scale Upland Crops . Journal of Biosystems Engineering, 2022, 47(3): 270-85.
7. SARKER K K, WANG X, LI H, et al. Development and Evaluation of Zone-till with Subsurface Fertilizer Applicator for Unpuddled Transplanting Rice Cultivation . Ama-Agricultural Mechanization in Asia Africa and Latin America, 2015, 46(2): 7-12.
8. DU J, YANG Q J, XIA J F, et al. DISCRETE ELEMENT MODELING AND VERIFICATION OF AN OUTER GROOVE WHEEL FERTILIZER APPLICATOR WITH HELICAL TEETH . Transactions of the Asabe, 2020, 63(3): 659-65.
9. HU H, LIU G, HUANG J, et al. INVESTIGATION OF THE INFLUENCING FACTORS OF FERTILIZER DROPPING TIME FOR A VARIABLE-RATE FERTILIZER APPLICATOR . Applied Engineering in Agriculture, 2022, 38(5): 797-806.
10. ZHANG M, TANG Y, ZHANG H, et al. Parameter Optimization of Spiral Fertilizer Applicator Based on Artificial Neural Network. Sustainability, 2023, 15(3).
11. YANG Y, CHEN Z, MA F, et al. DESIGN AND TEST OF KEY COMPONENTS OF 3ZFS-520 INTERTILLAGE DEEP FERTILIZER APPLICATOR . Inmateh-Agricultural Engineering, 2019, 59(3): 111-8.
12. YANG Y, CHEN Z, MA F, et al. DESIGN AND TEST OF KEY COMPONENTS OF 3ZFS-520 INTERTILLAGE DEEP FERTILIZER APPLICATOR . Inmateh-Agricultural Engineering, 2019, 59(3): 111-8.
13. ZHANG J, LIU G, HUANG J, et al. A STUDY ON THE TIME LAG AND COMPENSATION OF A VARIABLE-RATE FERTILIZER APPLICATOR . Applied Engineering in Agriculture, 2021, 37(1): 43-52.
14. Yang, Y.; Zhang, S.L.; Yang, X.Y.; Sun, B.H. Effect of Long-term Fertilization on Wheat yield, Nutrient Use Efficiency and Nutrient Balance in Rainfed Lou Soil. *Chinese journal of soil science*. **2017**, (05).
15. IQBAL, ACHMAD M, SAPSAL M T, et al. Organic fertilizer applicators design for supporting rice production; proceedings of the 1st International Conference on Global Issue for Infrastructure, Environment and Socio-Economic Development (IC-GIESED), Hasanuddin Univ, Post Grad Sch, Makassar, INDONESIA, F 2019Aug 30-Sep 01, 2018 . 2019.
16. Cartner J O . Ditching apparatus with improved ditching blade: US, doi:US4612715 A[P]. 1986.
17. Kazuhisa M. DITCHING MACHINE FOR RIDING:, JP2009167722A[P]. 2009.
18. Saeys, W.; Engelen, K.; Ramon, H. An automatic depth control system for shallow manure injection, Part 1: Modelling of the depth control system. *Biosystems Engineering*. **2007**, *98(2):146-154*.
19. Xiao, H.G.; Zhao, Y.; Ding, W.Q.; Mei, S.; Han, Y.; Zhang, Y.; Song, Z.Y. Design and experiment on blade shaft of 1KS60-35X type orchard double-helix trenching and fertilization machine. *Journal of Agricultural Engineering.* **2017**, *(10),32-39*.
20. Zhang, H.J.; Xu, C.B.; Liu, S.X.; Jiang, H.; Zhang, C.F.; Wang, J.X. Design and Experiment of Orchard Double Row Ditching-fertilizer Machine with Automatic Depth. *Transactions of the Chinese Society for Agricultural Machinery. Journal of Agricultural Machinery.* **2021**, *(01), 62-72.*
21. Shen, C.J.; Zhang, L.X.; Zhou, Y.; Dai, Y.M.; Li, F.; Zhang, J. Kinematic analysis and tests of the insertion mechanism of a self-propelled orchard gas explosion subsoiling and fertilizing machine. *Journal of Agricultural Engineering.* **2022**, *(01), 44-52*.
22. ZENG S, TAN Y, WANG Y, et al. Structural design and parameter determination for fluted-roller fertilizer applicator [J]. International Journal of Agricultural and Biological Engineering, 2020, 13(2): 101-10.
23. Zhao, L. Simulation and experiment of a spiral fertilizer discharger for mango fertilizer application machine based on discrete elements (Master's thesis, Kunming University of Technology).2019
24. Wai, G. J.; Qi, B.; Jiao, W.; Shi, S.; Jian, S.C. Design and Experiment of Mechanical Forced Fertilizing Device for Paddy Field . *Journal of Agricultural Machinery.* **2020**, *(S1), 154-164.*
25. XIAO,W.L.; LIAO,Y.T.;SHAN,Y.Y.;LI,M.L.; WANG,L.;LIAO,Q.X.Design and Experiment of Quad-screw Double-row Fertilizer Apparatus for Rape Seeding Machine.*Transactions of the Chinese Society for Agricultural Machinery*,**2021,***52(11):68-77.*
26. Yang,W.W.;Fang,L.Y.;Luo,X.W.;Li,H.;Ye,Y.Q.;Liang,Z.H.Experimental study of the effects of discharge port parameters on the fertilizing performance for fertilizer distribution apparatus with screw*.Transactions of the Chinese Society of Agricultural Engineering (Transactions of the CSAE)*,**2020**,*36(17):1-8.*
27. LIU,H.N.;LI,T.;,ZHAO,H.Y.;LI,J.;LEI,X.L.;REN,W.J.Design and experiment of screw-type feeding device of air-assisted centralized fertilizer application device.*Journal of China Agricultural University*,**2021***,26(08):150-161.*
